# Radish and Spinach Seedling Production and Early Growth in Response to Struvite Use as a Phosphorus Source

**DOI:** 10.3390/plants13202917

**Published:** 2024-10-18

**Authors:** Giannis Neofytou, Antonios Chrysargyris, Maria G. Antoniou, Nikolaos Tzortzakis

**Affiliations:** 1 Department of Agricultural Sciences, Biotechnology and Food Science, Cyprus University of Technology, 3603 Limassol, Cyprus; 2 Department of Chemical Engineering, Cyprus University of Technology, 3603 Limassol, Cyprus

**Keywords:** phosphorus, struvite, nutrient recovery, peat, vegetable crops

## Abstract

To sustain the increasing needs of a rapidly growing population, agriculture has relied on the use of synthetic fertilizers to intensify its production. However, the economical, environmental and health impacts associated with their use have raised significant concerns, especially given the scarcity of phosphorus. Utilizing nutrient-recovered materials like struvite can enhance circularity in agriculture and reduce its reliance on synthetic fertilizers. The objective of this study was to assess the implementation of struvite as a complete substitute to triple superphosphate, for radish and spinach seedling production and early growth, with or without supplementary fertigation. In addition, two rates of struvite were examined (0.68 and 1.37 g L^−1^ substrate) to evaluate its solubility. In the germination of radish, struvite had similar performance with conventional fertilization, while in spinach, the use of struvite decreased mean germination times. Both plants maintained comparable growth, chlorophyll content and antioxidant capacity when struvite was used, in comparison to conventional fertilizers. However, higher struvite rates under un-fertigated conditions significantly increased the chlorophyll b and total chlorophylls in the spinach, while phenolics and flavonoids decreased, contingent on the fertigation applications. In the radish, struvite maintained similar MDA and H_2_O_2_ levels to conventional fertilization, while decreases occurred in the spinach, with the application of ST1 under un-fertigated conditions, compared with conventional fertilization. The P and N contents of the plants were also affected, though these effects varied depending on the plant species, fertigation applications and struvite rates. This variance can be attributed to the characteristics of struvite, the plant species and the cultivation practices. The results of this study suggest that struvite can be successfully implemented in seedling production, establishing significant prospects for its commercialization and use in nurseries.

## 1. Introduction

The global population is rapidly growing and is expected to reach 9.4 to 9.7 billion by 2050 [1]. This trend commands significant increases in food availability, and thus agricultural crop production, which is directly linked with a greater use of inorganic fertilizers and natural resources [2]. While the use of synthetic fertilizers has enabled the intensification of crop production, it also presents several environmental consequences, in the form of water pollution through runoff, eutrophication and soil degradation and pollution [3]. Synthetic fertilizers are also responsible for human health adversities, as they may produce food high in nitrates and can contain toxic pollutants such as heavy metals, which are linked to various health issues [4].

Phosphorus (P) is a significant macronutrient for plant growth and production, and it is expected to decline in availability as phosphate rock reserves are non-renewable [5]. To sustain the increasing needs of agricultural crop production, P input has almost doubled over the past decade [6], while current methods of improving P use efficiency remain ineffective [7]. Long-term application of inorganic P fertilizers results in inefficient utilization of limited P supplies, and it can result in P build-up in soil, commonly known as legacy P, which is not immediately available to plants [8]. Additionally, excessive and continuous applications of fertilizers (e.g., inorganic fertilizers, manure, municipal wastes) may lead to P losses via leaching, resulting in the eutrophication of waterbodies [9]. P losses are expected to rise under climate change by up to 30% over the next 20 years, with analyses indicating that agriculture must reduce its use of P inputs by 20–80% to alleviate this effect [7]. It is thus important to maintain effective nutrient management and recovery strategies, by reusing nutrients in the form of organic materials derived from various sources [10,11]. Their use as partial or full substitutes to fertilizers, especially in intense cultivation systems (i.e., greenhouse crops and hydroponics) can alleviate the concerns over the use of synthetic fertilizers, while also maintaining adequate crop production to sustain the needs of the world population [12,13].

Struvite, produced from nutrient-rich wastes, presents a viable alternative to fertilizers, as it contains large sums of phosphate (${PO}_{4}^{3-}$), as well as ammonium nitrogen (${NH}_{4}^{+}$-N) and magnesium (Mg) [6,14]. Also known as magnesium ammonium phosphate $\left[Mg{NH}_{4}({PO}_{4}{)}_{3}\cdot 6H_{2}O\right]$, struvite is produced with equimolecular concentrations of ${PO}_{4}^{3-}$, ${NH}_{4}^{+}$ and Mg^2+^, under an alkaline pH [15]. Other parameters such as the temperature, stirring speed and presence of other ions such as K^+^ and Ca^2+^ influence the precipitation of struvite [11,16]. Researchers have highlighted the efficacy of struvite as a slow-releasing fertilizer [17], with a low salt index and low contents of heavy metals or pathogens [14]. In fact, the introduction of struvite in crop production systems maintained or improved the growth of the chili pepper (*Capsicum annuum* L.), cucumber (*Cucumis sativus* L.) [18], tomato (*Lycopersicum esculentum* L.) [19] and Italian ryegrass (*Lolium multiflorum* Lam.) [20]. Furthermore, the slow-releasing properties of struvite are significant for potted plant and seedling production, as irrigated water is constantly drained [21], as observed for zucchini (*Cucurbita pepo* L.) seedling production [22].

Although various research was conducted to assess the use of struvite as a fertilizer, most of it was conducted in soil-based cultivation systems. Recently, a small number of studies explored its use in soilless cultivation systems, with varying results. Specifically, El-Nakhel et al. [23] examined the use of struvite in the hydroponic cultivation of lettuce, which resulted in comparable performance to the control (NPK) fertilization. Similar results were obtained by Arcas-Pilz et al. [24] for the cultivation of the common bean (*Phaseolus vulgaris* L.) and by Halbert-Howard et al. [25] for tomato cultivation (*L. esculentum* L.). Furthermore, a seed germination study conducted by Melito et al. [22] showed that struvite had comparable performance to traditional fertilizers in terms of its germination rate, fresh and dry biomass production, with the particle size and feedstock of the produced struvite playing a pivotal role in its use as a nutrient supply alternative. In contrast, some studies reported a negative result with the use of struvite. In effect, in lettuce [26] and the common bean [24], growth and yield were impaired, mainly due to the lack of nutrition in the form of supplementary synthetic fertilization, higher P requirements due to the presence of rhizobia and reduced struvite dissolution. Therefore, struvite application needs to be tested individually for different crops and tailored based on the crop mineral needs, crop tolerance to saline and other abiotic stress conditions, struvite composition and release properties, struvite application methods, and environmental conditions during crop production.

Based on the above, this study assessed the impact of struvite produced from the anaerobically digested effluent of livestock waste, as a complete substitute to triple superphosphate (TSP), on the germination and early growth of radish (*Raphanus sativus* L.) and spinach (*Spinacia oleracea* L.), two species with different edible parts. Struvite was used at two rates (0.68 and 1.37 g L^−1^ substrate) to assess its availability, and it was also supplemented with a fertigation solution made up of conventional fertilizers, to assess its effectiveness at early stages of plant development and, after the experiment’s conclusion, on the germination rate, plant growth, nutrient accumulation, antioxidant capacity and stress responses.

## 2. Results

### 2.1. Effects of Base Fertilization and Supplementary Fertigation on Radish and Spinach Germination and Early Growth

Table 1 presents a summary of the effects of the base fertilization and supplementary fertigation and their interaction on the germination, growth, physiology, stress response and mineral status of radish and spinach seedlings. Fertilization significantly affected the radish DM, SPAD, leaf N, root Na, stomatal conductance, total phenolics, antioxidant capacity (DPPH, FRAP, ABTS), flavonoids, leaf and root P, and leaf K and Na. Supplementary fertigation significantly influenced the stomatal conductance, leaf N, P and Na, root K, and Na, growth-related parameters (height, stem diameter, leaf number, leaf DM, radish FW, leaf and lateral root FW), total phenolic content, and antioxidant capacity (DPPH, FRAP, ABTS). Finally, their interaction significantly affected the stem diameter, total Car:total Chl, leaf N, P and K, root P, K and Na, plant height and radish DM, stomatal conductance, total phenolics, and antioxidant capacity.

Fertilization significantly influenced the spinach mean germination time, chlorophyll fluorescence, Chl a, Chl b, total Chl, total Car, Chl a:Chl b ratio, leaf N, P and Na, leaf DM, antioxidants, flavonoids, H_2_O_2_, and leaf N. Supplementary fertigation impacted plant growth (plant height, leaf number, FW per pot and plant), chlorophyll fluorescence and leaf N, P and Na, plant growth physiological parameters (chlorophylls, Chl a:Chl b, total Car:total Chl), ABTS, and flavonoids. Finally, their interaction affected the total FW per pot, leaf DM, leaf number, chlorophyll fluorescence, Chl a, Chl b, total Chl, total Car and leaf N and P, flavonoids, and H_2_O_2_.

### 2.2. Radish Study

#### 2.2.1. Radish Seed Germination and Plant Growth

The results regarding germination (Figure S2) revealed that the radish seeds emerged at 3 DAS, while no further emergence was observed after 13 DAS. The application of supplementary fertigation stimulated seed emergence, as all treatments, excluding Fert+S, achieved a 100% emergence rate (Figure S3).

The impacts of the examined applications on the radish growth-related parameters are presented in Table 2. Generally, fertigation increased plant height; the highest and lowest were observed for the NoFert+S and NoFert treatments, respectively, demonstrating a 170% increase. For non-fertigated treatments, ST1 had the highest plant height, demonstrating a 47% increase from the NoFert treatment, and a 10% increase from the Fert and ST2 treatments, while ST2 demonstrated equivalent results to Fert. The opposite was observed for fertigated treatments, as Fert+S had no significant difference with ST2+S. Similarly, for the stem diameter, it was mostly higher for plants subjected to fertigated treatments. Furthermore, the application of Fert, ST1 and ST2 led to an increase in the stem diameter, as compared with the NoFert treatment, while the application of supplementary fertigation mostly nullified these increases, similar to the leaf number.

The leaf FW was mostly impacted by the application of supplementary fertigation, excluding treatment ST1+S, exhibiting equivalent results to its non-fertigated counterpart. A similar result was observed for the leaf DM content of leaves, as well as the radish FW, with the application of base fertilization not being a determining factor. On the contrary, the DM of the radish was mostly lower in the fertigated treatments, while an improvement was noted with the application of ST1+S, compared with the application of NoFert+S and Fert+S. Finally, the lowest FW of the lateral roots was observed for the ST2 treatment, and the highest for NoFert+S and ST2+S.

#### 2.2.2. Radish Physiological Parameters

The SPAD was increased with the ST2+S application by up to 43% compared with the other treatments, excluding ST1+S, while NoFert application resulted in the lowest SPAD. Furthermore, an increase in the chlorophyll fluorescence was noted with the application of ST1+S, while stomatal conductance was mostly positively influenced by the application of supplementary fertigation, excluding the NoFert treatment (Table 3). Additionally, no significant differences were found among the base fertilizer applications regarding the plant chlorophyll (Chl a, Chl b, total Chl) and carotenoid contents, although supplementary fertigation stimulated their increase. Regarding the Chl a:Chl b ratio, the lowest and highest were observed for the ST1 and Fert treatments, respectively, while the total Car:total Chl ratio was the lowest for the ST1 and ST2 treatments, and the highest for the NoFert treatment (Table 3).

#### 2.2.3. Radish Stress Indicators, Total Phenolics, Flavonoids and Antioxidant Capacities

**Figure 1:**
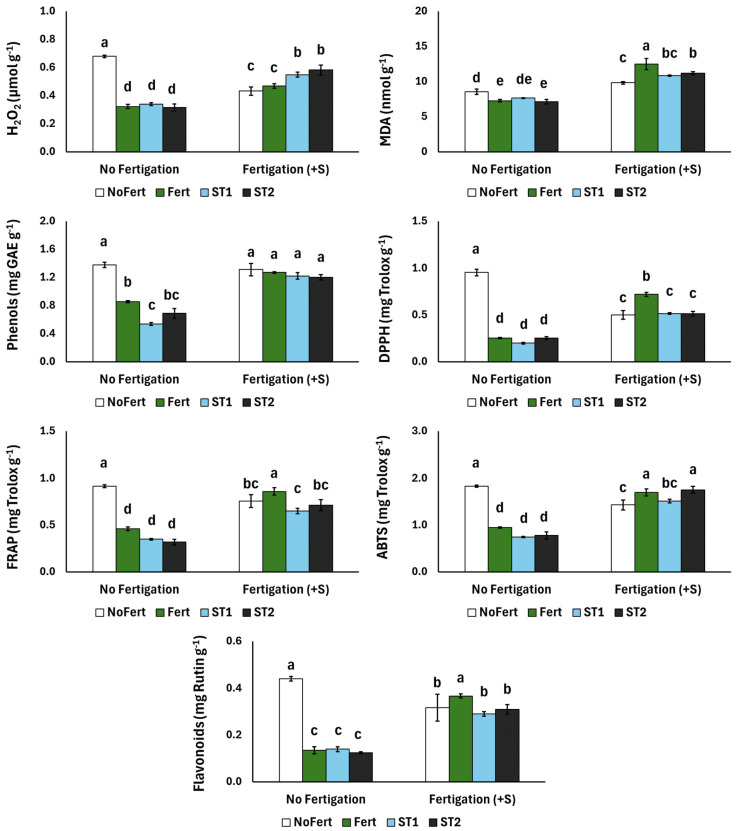
The effects of fertilizer application (NoFert, Fert, ST1 and ST2) and supplementary fertigation (+S) on the H_2_O_2_, MDA, content of total phenols, antioxidant capacity (FRAP), radical scavenging activity (DPPH and ABTS) and flavonoids of radish plants grown in peat. Significant differences (*p* < 0.05) among the treatments are indicated by different letters above the vertical bars. Values are means (±SE) of six replicates for each treatment.

Figure 1 presents the stress response (H_2_O_2_ and MDA), total phenolics, flavonoids and antioxidant capacities of the radish. Regarding H_2_O_2_, the highest production was observed for the NoFert treatment, which was subsequently lowered with the use of supplementary fertigation. Furthermore, the applications of Fert, ST1 and ST2 lowered H_2_O_2_, compared with the NoFert treatment, while the supplementary fertigation increased H_2_O_2_ among these treatments. Finally, the MDA was increased with supplementary fertigation, while the application of Fert and ST2 generally lowered the MDA, compared with NoFert, with fertigation having the opposite effect. The total phenolics in the radish generally decreased with the application of Fert, ST1 and ST2, compared with NoFert, while no changes were found among fertigated treatments. A similar trend was observed for the flavonoids and antioxidant capacities (assayed by DPPH, FRAP and ABTS), while supplementary fertigation increased the contents of flavonoids and antioxidants, excluding the NoFert treatment.

#### 2.2.4. Radish Leaf and Root Nutrient Content

The mineral content of the above-ground radish and its root tissue is shown in Table 4. As expected, the N content of leaves was lower in plants grown with no fertilization (NoFert), compared with the Fert, ST1 and ST2 treatments, while all treatments experienced an increase in leaf N content with the application of supplementary fertigation. The highest N content in leaves was found in the ST2+S treatment. Additionally, the P content was significantly lower in NoFert plant leaves, while the highest was observed in Fert+S. Interestingly, the application ST2+S achieved low P accumulation in the leaves, an effect similar to the P content of roots, although the highest root P content was observed for the ST2 treatment. Regarding roots, the P and K contents were the lowest in NoFert, and they were the highest in Fert+S for P and in ST1 for K. Furthermore, a significant decrease in K was observed in plants treated with ST1+S, compared with their non-fertigated counterpart. A similar phenomenon was observed for the K content of the roots. Finally, the application of Fert caused the highest accumulation of Na in leaves, while the lowest was observed for the ST2+S treatment. In the roots, Na was the highest for the ST2 treatment, while the lowest was for its fertigated counterpart.

### 2.3. Spinach Study

#### 2.3.1. Spinach Seed Germination and Plant Growth

The application of struvite (ST1 and ST2) stimulated seed emergence, as the seed emergence of these treatments was increased by up to 54.5%, as compared with the control treatment (NoFert), with the highest emergence rate being observed for the ST2+S treatment (Figure S4). Regarding the mean germination time, the highest was observed for the NoFert treatment, and the lowest for the ST2 and ST2+S treatments (Figure S5).

The impacts of the examined applications on spinach growth-related parameters are presented in Table 5. Supplementary fertigation generally increased plant height, and the highest values were observed for the ST2+S treatment, demonstrating increases of up to 205% and 89%, respectively. The FW of the spinach plants per pot was mostly increased by the application of supplementary fertigation. A similar result was observed for the FW per plant, as the impact of base fertilization did not have a significant effect. On the contrary, the DM of leaves was significantly higher in the NoFert treatment.

#### 2.3.2. Spinach Physiological Parameters

Selected physiological parameters are presented in Table 6. The SPAD was the lowest in plants subjected to the ST1 treatment and the highest in NoFert+S, ST1+S and ST2+S. Furthermore, chlorophyll fluorescence was decreased with the application of NoFert, while no significant differences were observed between the other treatments. Additionally, Chl a was the lowest in NoFert and ST1, and, similarly, NoFert-treated plants had the lowest Chl b and total Chl (Table 6). Finally, the Chl a:Chl b and total Car:total Chl ratios were the highest for the NoFert treatment, while the lowest were for the ST1+S and ST2+S treatments.

#### 2.3.3. Spinach Stress Indicators, Total Phenolics, Flavonoids and Antioxidant Capacities

**Figure 2:**
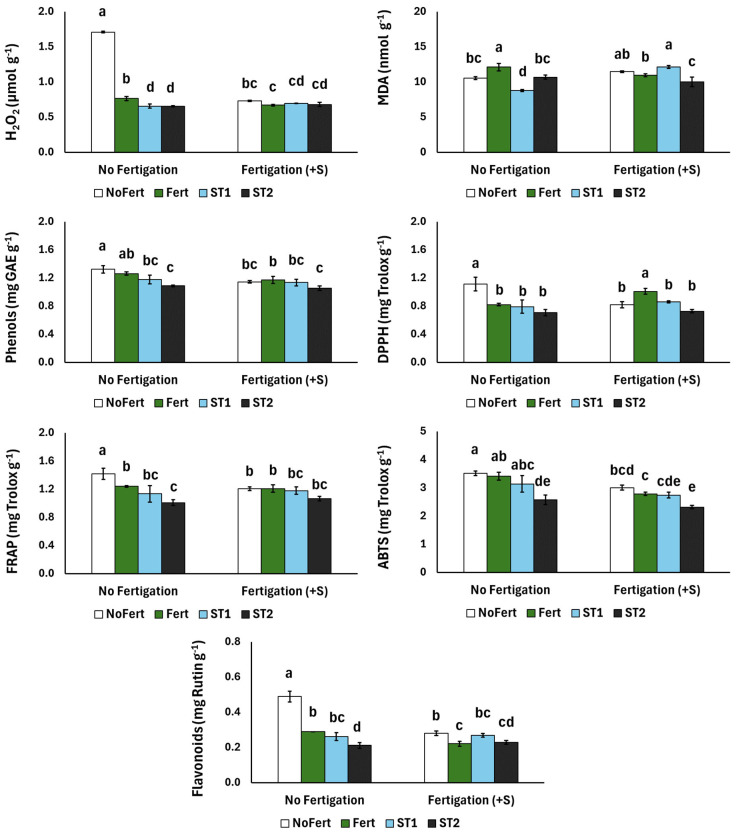
The effects of fertilizer application (NoFert, Fert, ST1 and ST2) and supplementary fertigation (+S) on H_2_O_2_, MDA, content of total phenols, antioxidant capacity (FRAP), radical scavenging activity (DPPH and ABTS) and flavonoids of spinach plants grown in peat. Significant differences (*p* < 0.05) among the treatments are indicated by different letters above the vertical bars. Values are means (±SE) of six replicates for each treatment.

Figure 2 shows the spinach plants’ stress responses (H_2_O_2_ and MDA), total phenolics, antioxidant capacities and flavonoids. H_2_O_2_ production was the highest with the application of NoFert, although this effect was suppressed with supplementary fertigation. Furthermore, the applications of Fert, ST1 and ST2 lowered H_2_O_2_ values, compared with the NoFert treatment, while supplementary fertigation decreased H_2_O_2_ for the Fert treatment. Finally, while the MDA in the spinach was decreased with supplementary fertigation for the Fert treatment, the opposite was observed for the ST1 treatment. Total phenolics were decreased with the application of ST2, compared with NoFert, while no significant differences were found between ST2 and ST2+S, which had the lowest phenolic production. Furthermore, flavonoids and antioxidant capacities (DPPH, FRAP and ABTS) exhibited an increase with the NoFert application, while the application of ST2 led to a decrease in FRAP, ABTS and flavonoids, regardless of fertigation.

#### 2.3.4. Spinach Leaf Nutrient Content

The mineral content of the spinach leaves is shown in Table 7. As expected, the N content of leaves was lower in plants grown with NoFert, with increases observed in the Fert, ST1 and ST2 treatments. Supplementary fertigation led to an overall increase in leaf N content. Interestingly, P was significantly lowered with the application of Fert, ST1 and ST2, compared with the NoFert treatment, although supplementary fertigation ameliorated this response, with ST2+S achieving the highest P content. K was significantly lower in the Fert treatment and the highest in the ST2 treatment. Finally, Na was the highest in the NoFert treatment and the lowest in the ST2 treatment.

## 3. Discussion

The recovery of P from waste streams is especially relevant in agriculture, as it can provide a substitute for conventional P fertilizers, which are sourced by non-renewable phosphate deposits [27]. Struvite is a valuable material for supporting a circular approach in agriculture, confirmed by life cycle analysis in hydroponics, which showed positive results in various impact categories, including global warming, freshwater eutrophication, mineral resource scarcity and terrestrial acidification, with deviations related to struvite quantity and produce yield [12].

Struvite has demonstrated high efficacy as a slow-release fertilizer in soil-based cultivation systems [28]. However, few studies report on its use in soilless cultures [29,30], and even fewer report on its use in seedling production [22]. Given the need for targeted and accurate fertilization in nurseries, and the rising costs of fertilizers [31,32], this study was conducted to evaluate struvite as a fertilizer constituent, in the germination and early growth of radish and spinach, focusing on the rates of struvite used and fertigation as a supplement to base fertigation.

### 3.1. Seed Germination Study

In seedling production, the germination process was evaluated through a series of germination metrics, including seed emergence, mean germination time and phytotoxicity tests. These measurements are used to assess the overall success of the germination process [33] and can directly influence the operational efficiency and economical viability of a nursery. These assays are significant when a novel material is examined as a possible fertilizer substitute. Struvite, being a primary source of P, can influence seed germination and root development [34]. A study conducted by Melito et al. [22] revealed that struvite from different feedstocks performed similarly to conventional fertilization at base fertilization levels, in the germination time and rate of zucchini (*C. pepo* L.) seeds. These findings were corroborated in the current study on radish, as struvite performed similarly to conventional fertilization, in terms of mean germination time and germination percentage. Conversely, in the study of spinach, the use of struvite decreased mean germination times, an important finding, as germination speed is fundamental for the establishment and success of crops, associated with improved seed vigor [35]. Furthermore, rapid germination enables crops to withstand unfavorable growth conditions, enhancing their resilience to various stress factors [36]. Regarding the rate of struvite, reports have presented variable results. For example, Melito et al. [22] reported that the twofold increase in struvite significantly increased the mean germination time, while, Katanda et al. [37] reported that struvite did not cause significant emergence reductions in canola (*Brassica napus* L.) germination, regardless of the struvite rate. The results of the latter report are in line with the results of this study on radish. In contrast, the twofold increase in struvite significantly decreased the germination time of spinach seeds. These results highlight the variable effects of struvite rates, which are dependent on the struvite feedstock, plant species and fertilization regime. Finally, the germination percentage reported in the current study, (>70%) for radish and spinach, is in accordance with previous germination studies [38,39]. This indicates that struvite can serve as a viable alternative to conventional fertilization in seedling production, demonstrating no evidence of phytotoxicity.

### 3.2. Early Plant Growth

Adequate levels of P enhance various morphological traits, including plant height and above-ground plant dry matter [9]. Phosphorus is crucial in the early stages of growth, as the stored P in seeds begins to deplete, while its deficiency negatively affects root development, plant size, biomass and phenological development [40]. The study of Jama-Rodzeńska et al. [29] showed that struvite had a similar performance to TSP in lettuce (*L. sativa* L.) growth (i.e., fresh weight, leaf number), as indicated by other reports [23,41]. These are in line with the results of the present study, as both radish and spinach maintained similar plant growth attributes (height, fresh biomass production, leaf number) with the application of struvite at both rates (ST1, ST2), compared with the application of conventional fertilization. Similar findings were reported by Melito et al. [22] on zucchini seedlings, and they stressed the influence of struvite quality on its solubility. Interestingly, in the present study, the ST1+S application produced a lower radish plant height, stem diameter and biomass, compared with the rest of the fertigated treatments. In contrast, under supplementary fertigation, spinach growth was generally comparable with the application of the base rate of struvite and conventional fertilization, while ST2+S resulted in increased plant height, leaf number and fresh weight. This was previously confirmed in lettuce (*L. sativa* L.) and common bean (*Phaseolus vulgaris* var. Pongo) growth, in soilless cultivation [42,43], although Talboys et al. [5] highlighted the importance of supplementary fertilization, especially in early plant growth, due to the low solubility of struvite.

### 3.3. Plant Physiology

Fertilizers affect the chlorophyll content of plants, especially N. Moreover, imbalanced P nutrition may cause leaf chlorosis, which may correspond to lower chlorophyll and carotenoid contents [44]. In the present study, struvite did not cause decreases in the chlorophylls (a, b, total) and carotenoids of radish and spinach leaves, compared with conventional fertilization, while notable decreases were caused by the absence of supplementary fertigation. In contrast, Jama-Rodzeńska et al. [29] and Ramut et al. [45] reported increased chlorophyll contents with the application of struvite, compared with TSP, as struvite provided higher amounts of N. The present study did not observe the same results, as all fertilization treatments had equal N concentrations. In addition, Melito et al. [22] observed increased SPAD with the twofold increase in struvite, mirroring the results of ST2+S of the current study. Struvite also has substantial amounts of Mg [46] which may influence the synthesis of chlorophyll, as Mg functions as the central atom of the chlorophyll molecule [47]. In fact, in spinach, the use of ST2 significantly increased the chlorophyll b and total chlorophyll contents compared with base conventional and struvite fertilization. Finally, the chlorophyll a to chlorophyll b ratio, used to assess the response of plants to various stresses [29], as affected by struvite, closely matched the results obtained by conventional fertilization, in both the radish and spinach.

### 3.4. Total Phenolics, Antioxidants, Flavonoids and Stress Indicators

Vegetables such as the radish and spinach are regarded for their dietary importance, due to their abundance of various health-promoting phytochemicals (e.g., phenolic and antioxidants). Their health benefits include antioxidant, anticancer, hypoglycemic and anti-obesity activities [48], indicating their functional roles as fresh produce. Previous studies have shown that struvite performed similarly to conventional fertilization, in terms of polyphenol and antioxidant production. Specifically, Nicastro et al. [49] observed that struvite did not elicit significant differences compared with the control treatment, regarding polyphenols and ABTS in lettuce, being in accordance with the present findings. Similarly, Melito et al. [22] observed no significant differences in terms of the total phenolics, between struvite and conventional fertilization. Stable total phenolics and antioxidant activity entail that plants under struvite fertilization are not experiencing significant stress that would trigger the production of antioxidant compounds. Interestingly, in the radish, the total phenolics revealed a decreased trend with the application of ST1, compared with Fert. Additionally, under fertigated conditions, flavonoids were decreased with the application of ST1+S and ST2+S, compared with Fert. Contrasting results were obtained in the study of spinach, as the application of ST2 decreased total phenolics, antioxidants (FRAP, ABTS) and flavonoids, compared with conventional fertilization. The variance in results exhibited in the current study and available literature can be attributed to the differences in plant species, cultivation methods, edible parts (leaves versus roots) and agronomic applications. Furthermore, Nicastro et al. [49] reported that the use of struvite in the soilless cultivation of lettuce resulted in a stress response (in terms of MDA and H_2_O_2_) similar to that of plants grown with conventional fertilizers. This was corroborated in the current study for radish under un-fertigated conditions. Under fertigated conditions, the H_2_O_2_ in the radish leaves was significantly increased with the application of struvite, while a notable decrease was observed in MDA, indicating reduced lipid peroxidation and cellular damage, compared with conventional fertilization. This suggests that the plant responded effectively to stress, or that H_2_O_2_ was not yet reduced via the detoxification of antioxidant enzymes [50]. Further investigation is required to evaluate the effects of antioxidant enzymes (e.g., superoxide dismutase—SOD, catalase—CAT, peroxidase—POD, ascorbate peroxidase—APX). Finally, struvite decreased the H_2_O_2_ in the spinach under un-fertigated conditions, while the MDA was only decreased with the application of struvite at the base rate. Previous research on the use of recovered nutrients has shown a variable crop stress response. With the application of nitrified urine, Jurga et al. [51] found increased H_2_O_2_ and lipid peroxidation in lettuce, as no extra nutrients were applied. The authors linked this stress response with the reduced P content. Such derivatives are also high in Na, which may cause stress, leading to the activation of enzymes to scavenge reactive oxygen species (ROS). This was not evident in the current experiment, as struvite is characterized by low contents of Na, and was evidenced by the Na content of plants [51].

### 3.5. Plant Nutritional Status

Struvite, applied as a fertilizer, is characterized by substantial amounts of P, N and Mg, and it is able to completely substitute conventional P sources [25]. Struvite characteristics, including dissolution, are related to the availability of these nutrients and can be influenced by cultivation conditions, such as the substrate pH [12]. Previous reports exhibited the effect of struvite on the P content of leaves [28,42], related to struvite quantity and dissolution [12]. Several studies suggest a variability in P uptake; higher P accumulation in wheat (*Triticum aestivum* L.) was reported by Zhang et al. [52], while in the common bean (*P. vulgaris* var. Pong) the P content of plants was lower, compared with conventional fertilization, as struvite remained largely undissolved [43]. In the present study, the leaf P content was influenced by the rate of struvite applied and varied among the radish and spinach. Specifically, ST2 and ST2+S increased the P content of the radish and spinach leaves, respectively, over conventional fertilization, an effect observed in previous studies [42] associating struvite rates with P accumulation. This was also evident in the P content of the radish roots, as an increase was observed with the applications of ST2 and ST1+S. Differences in the accumulation of P based on the rate of struvite may be related to the slow release, which limits the supply of P [5]. Concerning the content of N in leaf tissue, an increase was found in the radish, with the application of the increased rate of struvite under fertigated conditions (ST2+S), compared with conventional fertilization (Fert+S). This is in line with previous reports [23,52], as struvite provided higher N. In the spinach, N levels were not influenced by struvite, compared with conventional fertilization. In contrast, without fertigation, spinach exhibited increased K contents with the application of an increased struvite rate, compared with base struvite and conventional fertilization, as previously reported [29]. Potassium is crucial in plant growth, quality and physiology, but it is also vital for human health. Insufficient intake of K may result in health complications, making the biofortification of K in food more prevalent [53]. In contrast, hyperkalemia (elevated K levels in the bloodstream) is significant, particularly among chronic kidney disease patients [54]. Potassium accumulation depends on factors such as the cultivar, growth period, plant tissue and sampling time [55]. Sufficient levels of K in spinach range from 50 to 80 g kg^−1^ [56]. In the current study, the highest K (102.38 g kg^−1^) was observed with the use of ST2. While elevated, it remained consistent with previously reported values, ranging from 86.74 to 118.91 g kg^−1^ [57], in the spinach, cultivated hydroponically and harvested at 21 DAT.

## 4. Materials and Methods

### 4.1. Plant Material and Experimental Setup

The germination experiment using radish (*R. sativus* L.) and spinach (*S. oleracea* L.) was conducted at the experimental greenhouse of the Cyprus University of Technology in Limassol (CUT), from March to May 2024, for a total duration of 37 and 39 days after seeding (DAS), for the radish and spinach, respectively.

The current experiment consisted of 8 treatments per plant species. Each treatment had 10 replicates (pots) that were assigned to experimental units (trays), in a non-randomized design, having uniform climatic conditions in the greenhouse, due to the small surface area needed for the experimental setup. Different substrate mixtures were prepared with the use of peat and consisted of non-fertilized treatment (NoFert), conventional fertilizer treatment (FERT), with triple superphosphate (TSP, 0-46-0) as the source of P, and two treatments with struvite, as a source of P and N, at two different struvite rates (ST1 and ST2). Finally, the abovementioned treatments were repeated, and supplementary fertigation (electrical conductivity, EC: 1.5–1.8 mS cm^−1^) using conventional fertilizers (i.e., 20-20-20) was applied (supplementary fertigation: +S). All treatments, excluding the NoFert treatment, also included the use of ammonium nitrate and potassium sulfate as an adequate source of N and K, respectively (Table S1).

The struvite was produced at a pilot plant of CUT, located in Monagroulli, Limassol, at Armenis Farm Limited, from digestated effluent of anaerobically treated livestock waste. A Mg^2+^:NH_4_^+^-N:PO_4_^3−^-P molar ratio of 1.5:1:1 was used to achieve optimal struvite precipitation with a purity of over 90 wt % and an absence of pathogens and heavy metals [15].

### 4.2. Germination and Plant Growth Study

Radish and spinach seeds were sown in 0.5 L pots with the above-mentioned substrate media. For radish, one seed was sown per pot at a depth of 0.5 cm, while for spinach, 7 seeds were sown, at a depth of 0.5 cm and with adequate spacing to ensure placement uniformity. Then, the pots were topped off with the prepared medium (0.5 cm depth). Each treatment included 10 replications, with each replication consisting of a single pot, for each of the plant species examined. Irrigation frequency and quantity was based on the crop growth stage; pots were initially irrigated every two to three days, whereas at late stages, irrigation was conducted every one to two days. Emergence was monitored and recorded on a daily basis. The fluctuating temperature (°C) and relative humidity (%) are presented in Figure S1, averaging between 24.1 and 16.8 °C and 51.8 and 64.1% for day and night, respectively.

### 4.3. Plant Physiology

Prior to harvesting, the leaf fluorescence was measured (OS-30p, Opti-sciences, Hoddesdon, UK) after incubation in the dark for 15 min. The SPAD was also measured, using SPAD-502 Plus (Konica Minolta, Tokyo, Japan). Other measurements included the leaf number, plant height (cm), stem diameter (mm), above-ground fresh weight—FW (g) and total dry matter content—DM (%), primary root FW (g) and DM (%), and lateral root FW (g).

The leaf chlorophyll content (Chl a, Chl b and total Chl), and total carotenoids (total Car) were calculated following methanol extraction, and measurement of absorbance at 470, 653 and 666 nm (Multiskan GO, Thermo Fischer Scientific Oy, Finland), as described previously [58]. The results are expressed as mg g^−1^ FW.

### 4.4. Determination of Total Phenolics, Flavonoids, Antioxidants and Stress Indicators

Methanolic extracts of the leaf samples were used for the determination of the total phenolic content, by the Folin–Ciocalteu method, at 755 nm, as previously described [58]. The results are expressed as equivalents of gallic acid (GAE) per gram of fresh weight (mg of GAE g^−1^ FW), using a calibration curve with GA.

Additionally, the methanolic extracts were used to determine the antioxidant activity, by the ferric-reducing antioxidant power (FRAP) and the 2,2-diphenyl-1-picrylhydrazyl (DPPH) and 2,2′-azinobis-(3-ethylbenzothiazoline-6-sulfonic acid) (ABTS) methods, as previously described [58]. For the FRAP, reaction mixtures were measured at 593 nm, and for the DPPH, radical scavenging activity was measured at 517 nm. The ABTS was measured using an ABTS solution (Sigma-Aldrich, Taufkirchen, Germany) and the absorbance was measured at 734 nm against a blank sample. In all abovementioned protocols, Trolox ((±)-6-hydroxy-2,5,7,8-tetramethylchromane-2-carboxylic acid) was used as a positive control, and the results for antioxidant activities are expressed as Trolox equivalents (mg of Trolox g^−1^ FW).

Flavonoids were determined using the aluminum chloride colorimetric method, as previously described [59], by measuring the absorbance at 510 nm (Multiskan GO, Thermo Fischer Scientific Oy, Vanda, Finland). The results are expressed as rutin equivalents per g of fresh weight (mg Rutin g^−1^ FW).

Hydrogen peroxide (H_2_O_2_) levels and lipid peroxidation in terms of the malondialdehyde content (MDA) were evaluated as previously described [58]. H_2_O_2_ was determined by measuring the absorbance at 390 nm, and results are expressed as μmol of H_2_O_2_ per gram of fresh weight (μmol H_2_O_2_ g^−1^ FW). MDA was quantified by measuring the absorbance of the reaction mixtures at 532 nm and corrected at 600 nm for non-specific absorbance. The quantity of MDA was calculated using the extinction coefficient of 155 mM^−1^ cm^−1^, and results are expressed as nmol of MDA per gram of fresh weight (nmol MDA g^−1^ FW).

### 4.5. Determination of Plant Mineral Content

Elemental analysis was conducted using dried tissue from the leaves and radish roots, subsequently dry ashed at 550 °C and extracted by hydrochloric acid digestion (2 N HCl). Potassium (K) and sodium (N) measured by using a flame photometer (Lasany Model 1832, Lasany International, Panchkula, India) and phosphorus (P) by the molybdate–vanadate method. Nitrogen (N) determination was conducted with the Kjeldahl method (BUCHI, Digest automat K-439 and Distillation Kjelflex K-360, Flawil, Switzerland) [58]. The plant content of the macronutrients is expressed in g kg^−1^.

### 4.6. Statistical Analysis

The statistical analysis of the data was conducted using SPSS v. 26.0 program (IBM Corp., Armonk, NY, USA), presenting the data as the mean ± standard error (SE), after performing one- or two-way analysis of variance (ANOVA) and mean separation by the Duncan multiple range test (DMRT) at *p* < 0.05.

## 5. Conclusions

The results of the current study suggest that struvite can be successfully applied in spinach and radish seedling production, as a complete P substitute, establishing prospects for its commercial exploitation in nursery production. In the radish, struvite matched the performance of conventional fertilization, in terms of mean germination time and germination percentage, while in the spinach, the use of struvite drastically decreased mean germination times, which shortened the duration during seedling production. Struvite supported the growth of both radish and spinach. In the radish, the SPAD, chlorophyll fluorescence and stomatal conductance largely remained unaffected by the examined parameters, while the increased struvite rates in conjunction with supplementary fertigation increased these values. Similarly, increased struvite rates produced increased chlorophyll b and total chlorophylls in the spinach, while decreases occurred in the mean germination time of the seeds, total phenolic content, as well as the antioxidant activity of the spinach plants, assayed by the FRAP and ABTS. This was not as apparent in the radish, as the antioxidant activity largely remained unaffected. Regarding stress indicators, in the radish, struvite maintained similar levels to conventional fertilization. In the spinach, however, H_2_O_2_ increased under un-fertigated conditions, while MDA decreased with the base struvite rate. In terms of plant nutrition, struvite application significantly increased the leaf P content of radish under un-fertigated conditions, although this was not apparent with the use of supplementary fertigation. In the spinach, however, without fertigation, leaves accumulated increased P with the application of ST1 under un-fertigated conditions. In contrast, with fertigation, ST2+S led to the highest leaf P content. Thus, the uptake of P varied depending on the plant species, fertigation application and struvite rate. Struvite was also shown to provide higher N availability for radish, as plants supplied with ST1+S and ST2+S had the highest N content. This variance in results is attributed to factors such as the characteristics of struvite, plant species, cultivation method and fertilization applied. Further research must be undertaken to investigate the specific properties of struvite that influence its effect across different plant species and cultivation practices, while also developing different fertilization strategies to improve its efficacy.

## Figures and Tables

**Table 1:** The effects of base fertilization (BF), supplementary fertigation (SF) and their interaction (BF × SF) on radish and spinach seedling growth, physiology, antioxidant capacity, stress response and nutrient content.

		Radish			Spinach	
	BF	SF	BF × SF	BF	SF	BF × SF
Mean germination time (d)	ns	ns	ns	*	ns	ns
Total seed emergence (%)	ns	ns	ns	ns	ns	ns
Plant number	–	–	–	ns	ns	ns
Plant height (cm)	ns	***	**	ns	***	ns
Stem diameter (mm)	ns	***	*	–	–	–
Leaf number	ns	***	ns	ns	***	*
Leaf FW (g)	ns	**	ns	ns	***	ns
Leaf DM (%)	ns	***	ns	**	**	***
Total FW pot^−1^ (g)	–	–	–	ns	***	***
Radish FW (g)	ns	***	ns	–	–	–
Radish DM (%)	*	ns	**	–	–	–
Lateral root FW (g)	ns	**	ns	–	–	–
SPAD	**	ns	ns	ns	ns	ns
Chlorophyll fluorescence (Fv Fm^−1^)	ns	ns	ns	*	*	*
Stomatal conductance (µmol m^−1^ s^−1^)	***	*	***	–	–	–
Chl a (mg g^−1^ FW)	ns	***	ns	*	***	***
Chl b (mg g^−1^ FW)	ns	***	ns	**	***	*
Total chlorophylls (mg g^−1^ FW)	ns	***	ns	*	***	**
Total carotenoids (mg g^−1^ FW)	ns	***	ns	*	ns	*
Chl a:Chl b	ns	ns	ns	*	***	ns
Total carotenoids: Total chlorophylls	ns	ns	*	**	***	ns
H_2_O_2_ (μmol g^−1^)	***	***	***	***	***	***
MDA (nmol g^−1^)	ns	***	**	ns	ns	*
Phenols (mg GAE g^−1^)	***	***	***	ns	ns	ns
DPPH (mg Trolox g^−1^)	***	***	***	*	ns	**
FRAP (mg Trolox g^−1^)	***	***	***	*	ns	ns
ABTS (mg Trolox g^−1^)	***	***	***	*	**	ns
Flavonoids (mg Rutin g^−1^)	***	***	***	***	***	***
Leaf N (g kg^−1^)	**	***	*	***	***	***
Leaf P (g kg^−1^)	***	***	***	*	***	**
Root P (g kg^−1^)	***	**	***	–	–	–
Leaf K (g kg^−1^)	***	ns	***	ns	ns	ns
Root K (g kg^−1^)	ns	*	***	–	–	–
Leaf Na (g kg^−1^)	***	*	ns	**	*	ns
Root Na (g kg^−1^)	**	***	***	–	–	–

***: significant at *p* < 0.001; **: significant at *p* < 0.01; *: significant at *p* < 0.05; ns: not significant, according to 2-way ANOVA. Fresh weight (FW); dry matter (DM); hydrogen peroxide (H_2_O_2_); malondialdehyde (MDA); 2,2-diphenyl-1-picrylhydrazyl (DPPH); ferric-reducing antioxidant power (FRAP); 2,20-azino-bis(3-ethylbenzothiazoline-6-sulphonic acid) (ABTS).

**Table 2:** The effects of fertilizer application (NoFert, Fert, ST1 and ST2) and supplementary fertigation (+S) on plant height (cm), stem diameter (mm), leaf number, leaves fresh and dry weight (g), radish fresh and dry weight, and lateral root fresh weight of radish plants grown in peat.

Fertilizer Application	Plant Height	Stem Diameter	Leaf No.	Leaf Fresh Weight	Leaf Dry Matter Content	Radish Fresh Weight	Radish Dry Matter Content	Lateral Root Fresh Weight
**NoFert**	8.38 ± 0.55 e	4.26 ± 0.08 d	5.00 ± 0.41 e	4.27 ± 2.77 b	5.69 ± 0.05 d	1.77 ± 0.55 d	5.69 ± 0.02 bcd	0.07 ± 0.02 cd
**Fert**	11.20 ± 0.93 d	5.67 ± 0.52 cd	6.00 ± 0.32 cde	3.94 ± 0.93 b	6.77 ± 0.56 bcd	1.79 ± 0.76 d	6.54 ± 0.03 a	0.08 ± 0.02 bcd
**ST1**	12.30 ± 1.24 cd	6.71 ± 0.50 c	6.60 ± 0.68 bcd	5.64 ± 1.21 b	6.13 ± 0.04 d	2.75 ± 0.82 d	5.52 ± 0.03 cd	0.07 ± 0.01 cd
**ST2**	11.20 ± 0.87 d	5.29 ± 0.79 cd	5.40 ± 0.81 de	3.32 ± 1.20 b	6.51 ± 0.09 cd	1.71 ± 1.08 d	6.11 ± 0.02 abc	0.06 ± 0.01 d
**NoFert+S**	22.60 ± 0.33 a	11.70 ± 0.07 a	8.40 ± 0.24 a	16.93 ± 0.92 a	8.99 ± 1.36 ab	32.75 ± 2.15 a	5.27 ± 0.23 d	0.16 ± 0.02 a
**Fert+S**	19.90 ± 1.00 b	10.82 ± 0.65 a	8.00 ± 0.32 ab	13.76 ± 0.92 a	10.12 ± 0.38 a	22.25 ± 3.93 b	5.58 ± 0.16 cd	0.13 ± 0.02 abc
**ST1+S**	14.30 ± 0.64 c	9.15 ± 0.28 b	7.40 ± 0.51 abc	5.94 ± 0.86 b	9.29 ± 0.51 a	10.62 ± 1.90 c	6.28 ± 0.19 ab	0.14 ± 0.04 ab
**ST2+S**	18.70 ± 0.92 b	11.67 ± 0.44 a	8.00 ± 0.32 ab	16.38 ± 1.69 a	8.76 ± 0.14 abc	30.26 ± 3.10 a	5.85 ± 0.25 bcd	0.16 ± 0.02 a

Values are the mean ± SE (*n* = 6). In each column, values followed by the same letter do not differ significantly at *p* < 0.05.

**Table 3:** The effects of fertilizer application (NoFert, Fert, ST1 and ST2) and supplementary fertigation (+S) on the SPAD, chlorophyll fluorescence (Fv Fm^−1^), stomatal conductance (µmol m^−1^ s^−1^), chlorophyll (Chl a, Chl b, Total Chl) and total carotenoid (total Car) content (mg g^−1^ FW), and Chl a:Chl b and total Car:total Chl ratios of radish plant leaves.

Fertilizer Application	SPAD	Chlorophyll Fluorescence	Stomatal Conductance	Chl a	Chl b	Total Chl	Total Car	Chl a:Chl b	Total Car: Total Chl
**NoFert**	33.75 ± 1.06 c	0.82 ± 0.01 ab	543.33 ± 91.71 c	0.42 ± 0.01 c	0.17 ± 0.00 c	0.59 ± 0.01 c	0.050 ± 0.000 bc	2.44 ± 0.00 ab	0.095 ± 0.005 a
**Fert**	38.28 ± 1.47 bc	0.82 ± 0.01 ab	606.67 ± 8.82 c	0.57 ± 0.06 bc	0.22 ± 0.01 bc	0.79 ± 0.07 bc	0.065 ± 0.005 bc	2.61 ± 0.18 a	0.085 ± 0.005 ab
**ST1**	40.65 ± 2.61 b	0.81 ± 0.02 b	538.33 ± 36.09 c	0.46 ± 0.03 bc	0.23 ± 0.03 bc	0.68 ± 0.01 bc	0.045 ± 0.015 bc	2.01 ± 0.38 c	0.065 ± 0.015 c
**ST2**	38.65 ± 2.70 bc	0.83 ± 0.01 ab	588.33 ± 72.59 c	0.38 ± 0.01 c	0.17 ± 0.01 c	0.55 ± 0.02 c	0.035 ± 0.005 c	2.30 ± 0.02 abc	0.065 ± 0.005 c
**NoFert+S**	41.65 ± 2.75 b	0.83 ± 0.00 ab	696.67 ± 12.02 bc	1.07 ± 0.02 a	0.49 ± 0.01 a	1.56 ± 0.02 a	0.130 ± 0.000 a	2.17 ± 0.01 bc	0.080 ± 0.000 abc
**Fert+S**	36.53 ± 1.91 bc	0.83 ± 0.00 ab	184.67 ± 13.48 d	0.88 ± 0.03 ab	0.42 ± 0.02 ab	1.30 ± 0.05 ab	0.097 ± 0.003 ab	2.12 ± 0.02 bc	0.073 ± 0.003 bc
**ST1+S**	42.43 ± 2.43 ab	0.84 ± 0.01 a	846.67 ± 63.60 ab	1.09 ± 0.04 a	0.50 ± 0.02 a	1.59 ± 0.06 a	0.123 ± 0.003 a	2.20 ± 0.02 bc	0.077 ± 0.003 bc
**ST2+S**	48.15 ± 0.69 a	0.83 ± 0.01 ab	900.00 ± 40.41 a	0.67 ± 0.28 abc	0.31 ± 0.13 abc	0.98 ± 0.41 abc	0.080 ± 0.035 abc	2.14 ± 0.01 bc	0.080 ± 0.000 abc

Values are the mean ± SE (*n* = 6). In each column, values followed by the same letter do not differ significantly at *p* < 0.05.

**Table 4:** The effects of fertilizer application (NoFert, Fert, ST1 and ST2) and supplementary fertigation (+S) on macronutrient content of leaf and root tissue (g kg^−1^) of radish plants grown in peat.

**Leaves**				
**Fertilizer** **Application**	**N**	**P**	**K**	**Na**
**NoFert**	35.00 ± 0.02 d	4.90 ± 0.02 d	28.41 ± 0.03 d	14.93 ± 0.04 c
**Fert**	43.83 ± 0.79 c	6.31 ± 0.10 c	49.78 ± 2.95 b	21.33 ± 1.38 a
**ST1**	46.05 ± 0.19 c	6.73 ± 0.41 c	55.59 ± 0.11 a	16.17 ± 1.00 bc
**ST2**	47.32 ± 0.57 c	9.22 ± 0.62 b	47.85 ± 0.02 bc	13.70 ± 1.48 c
**NoFert+S**	55.90 ± 1.60 ab	7.00 ± 0.24 c	46.28 ± 1.24 bc	14.62 ± 0.38 c
**Fert+S**	53.70 ± 0.66 b	13.25 ± 0.69 a	49.18 ± 1.01 b	18.03 ± 1.07 b
**ST1+S**	58.20 ± 2.35 ab	10.06 ± 0.42 b	44.69 ± 1.10 c	16.44 ± 0.66 bc
**ST2+S**	60.60 ± 2.80 a	6.45 ± 0.18 c	47.17 ± 1.26 bc	9.78 ± 0.66 d
**Roots**				
**Fertilizer** **Application**	**N**	**P**	**K**	**Na**
**NoFert**	nd	8.16 ± 0.01 c	47.62 ± 0.07 f	13.06 ± 0.03 bc
**Fert**	nd	8.05 ± 0.03 c	54.28 ± 0.05 de	12.14 ± 0.01 c
**ST1**	nd	8.81 ± 0.02 bc	67.37 ± 0.04 a	14.32 ± 0.01 b
**ST2**	nd	10.63 ± 0.02 a	56.22 ± 0.01 d	18.93 ± 0.02 a
**NoFert+S**	nd	6.80 ± 0.31 d	65.75 ± 2.32 ab	9.63 ± 0.83 d
**Fert+S**	nd	9.32 ± 0.30 b	61.38 ± 0.68 bc	10.31 ± 0.18 d
**ST1+S**	nd	10.23 ± 0.31 a	49.90 ± 0.90 ef	11.80 ± 0.13 c
**ST2+S**	nd	6.90 ± 0.31 d	57.84 ± 1.94 cd	7.33 ± 0.56 e

Values are the mean ± SE (*n* = 6). In each column, values followed by the same letter do not differ significantly at *p* < 0.05; nd: not detected due to limited dry tissue material.

**Table 5:** The effects of fertilizer application (NoFert, Fert, ST1 and ST2) and supplementary fertigation (+S) on plant number, plant height (cm), leaf number, leaf fresh weight per plant (g), total yield per pot (g) and dry matter content (%) of spinach plants grown in peat.

Fertilizer Application	Plant Number	Plant Height	Leaf No.	Plant Fresh Weight	Yield per Pot	Dry Matter Content
**NoFert**	5.40 ± 0.51	8.60 ± 0.40 e	7.00 ± 0.00 cd	0.84 ± 0.10 c	3.08 ± 0.31 e	12.82 ± 0.07 a
**Fert**	5.00 ± 0.00	13.80 ± 0.86 cd	7.60 ± 0.60 cd	2.32 ± 0.27 c	6.50 ± 0.36 cd	9.99 ± 0.40 b
**ST1**	5.00 ± 0.00	13.00 ± 0.76 d	6.40 ± 0.51 d	1.64 ± 0.31 c	4.84 ± 0.51 de	8.50 ± 0.16 d
**ST2**	5.00 ± 0.00	16.00 ± 0.35 c	7.00 ± 0.55 cd	2.68 ± 0.44 c	8.42 ± 0.76 c	8.39 ± 0.21 d
**NoFert+S**	4.80 ± 0.20	22.80 ± 0.92 b	9.40 ± 0.60 bc	7.42 ± 0.37 ab	30.12 ± 1.03 a	8.30 ± 0.05 d
**Fert+S**	4.80 ± 0.20	23.10 ± 1.26 b	10.80 ± 1.39 b	6.96 ± 1.13 b	23.36 ± 1.62 b	8.27 ± 0.10 d
**ST1+S**	5.00 ± 0.00	22.20 ± 0.73 b	11.20 ± 0.86 ab	6.34 ± 0.53 b	29.42 ± 0.95 a	9.55 ± 0.74 bc
**ST2+S**	5.00 ± 0.00	26.20 ± 1.20 a	13.20 ± 0.97 a	8.86 ± 0.94 a	28.34 ± 1.37 a	8.65 ± 0.23 cd

Values are the mean ± SE (*n* = 6). In each column, values followed by the same letter do not differ significantly at *p* < 0.05.

**Table 6:** The effects of fertilizer application (NoFert, Fert, ST1 and ST2) and supplementary fertigation (+S) on SPAD, chlorophyll fluorescence (Fv Fm^−1^), chlorophyll (Chl a, Chl b, total Chl) and total carotenoid (total Car) content (mg g^−1^ FW), and Chl a:Chl b and total Car:total Chl (µmol m^−1^ s^−1^) of spinach plant leaves grown in peat.

Fertilizer Application	SPAD	Chlorophyll Fluorescence	Chl a	Chl b	Total Chl	Total Car	Chl a:Chl b	Total Car: Total Chl
**NoFert**	26.03 ± 4.21 ab	0.74 ± 0.01 b	0.72 ± 0.01 c	0.22 ± 0.04 e	0.94 ± 0.05 e	0.17 ± 0.01 ab	3.43 ± 0.61 a	0.186 ± 0.024 a
**Fert**	27.18 ± 3.68 ab	0.82 ± 0.01 a	0.81 ± 0.02 bc	0.35 ± 0.02 d	1.15 ± 0.04 d	0.16 ± 0.00 ab	2.34 ± 0.13 b	0.140 ± 0.002 ab
**ST1**	19.23 ± 1.36 b	0.80 ± 0.00 a	0.71 ± 0.09 c	0.30 ± 0.03 de	1.01 ± 0.12 de	0.13 ± 0.02 bc	2.30 ± 0.31 b	0.132 ± 0.004 b
**ST2**	25.38 ± 2.07 ab	0.80 ± 0.00 a	0.93 ± 0.03 b	0.47 ± 0.03 c	1.40 ± 0.06 c	0.16 ± 0.01 ab	1.91 ± 0.18 bc	0.106 ± 0.011 bc
**NoFert+S**	30.20 ± 2.96 a	0.81 ± 0.00 a	1.13 ± 0.05 a	0.60 ± 0.03 b	1.73 ± 0.08 a	0.20 ± 0.01 a	1.96 ± 0.05 bc	0.122 ± 0.004 b
**Fert+S**	27.00 ± 2.51 ab	0.82 ± 0.01 a	0.89 ± 0.06 b	0.73 ± 0.04 a	1.62 ± 0.04 ab	0.08 ± 0.04 d	1.18 ± 0.07 c	0.039 ± 0.032 d
**ST1+S**	29.25 ± 3.02 a	0.81 ± 0.01 a	0.91 ± 0.03 b	0.60 ± 0.05 b	1.51 ± 0.04 bc	0.14 ± 0.01 abc	1.74 ± 0.05 bc	0.106 ± 0.009 bc
**ST2+S**	34.03 ± 3.60 a	0.81 ± 0.01 a	0.90 ± 0.04 b	0.70 ± 0.02 ab	1.60 ± 0.06 abc	0.09 ± 0.01 cd	1.26 ± 0.03 c	0.058 ± 0.004 cd

Values are the mean ± SE (*n* = 6). In each column, values followed by the same letter do not differ significantly at *p* < 0.05.

**Table 7:** The effects of fertilizer application (NoFert, Fert, ST1 and ST2) and supplementary fertigation (+S) on macronutrient content of leaf and root tissue (g kg^−1^) of spinach plants grown in peat.

Leaves				
Fertilizer Application	N	P	K	Na
**NoFert**	17.78 ± 0.13 c	10.53 ± 0.48 c	82.23 ± 4.74 cd	9.50 ± 0.61 a
**Fert**	46.96 ± 1.29 b	6.67 ± 0.39 e	78.95 ± 2.24 d	7.21 ± 0.18 bc
**ST1**	44.19 ± 0.83 b	8.72 ± 0.12 d	89.18 ± 3.34 bcd	7.82 ± 0.11 b
**ST2**	49.05 ± 1.81 b	6.21 ± 0.59 e	102.38 ± 5.35 a	5.50 ± 0.48 d
**NoFert+S**	57.52 ± 2.72 a	11.79 ± 0.57 bc	82.00 ± 2.07 cd	7.23 ± 0.34 bc
**Fert+S**	56.56 ± 1.25 a	11.47 ± 0.06 bc	91.57 ± 4.42 abc	6.93 ± 0.12 bc
**ST1+S**	55.58 ± 0.70 a	13.15 ± 1.08 ab	94.13 ± 2.04 ab	7.08 ± 0.33 bc
**ST2+S**	54.47 ± 0.93 a	14.12 ± 0.53 a	94.69 ± 3.36 ab	6.42 ± 0.31 cd

Values are the mean ± SE (*n* = 6). In each column, values followed by the same letter do not differ significantly at *p* < 0.05.

## Data Availability

The data used in this study are only available upon request.

## Supplementary Materials

The following supporting information can be downloaded at: https://www.mdpi.com/article/10.3390/plants13202917/s1, Table S1: Description of the treatments examined in this experiment. Figure S1: Average day and night temperature and relative humidity during the growth period. Figure S2: The effects of fertilizer application (NoFert, Fert, ST1 and ST2) and supplementary fertigation (+S) on daily seedling emergence (A,B) of radish seeds. Figure S3: The effects of fertilizer application (NoFert, Fert, ST1 and ST2) and supplementary fertigation (+S) on mean germination time (d) of radish seeds. Figure S4: The effects of fertilizer application (NoFert, Fert, ST1 and ST2) and supplementary fertigation (+S) on daily seedling emergence (A, B) of spinach seeds. Figure S5: The effects of fertilizer application (NoFert, Fert, ST1 and ST2) and supplementary fertigation (+S) on mean germination time (d) of spinach seeds. Figure S6: Photo comparison of radish growth between the examined treatments at end of the cultivation period. Figure S7: Photo comparison of radish growth between the examined treatments at end of the cultivation period. Figure S8: Photo comparison of spinach growth between the examined treatments at end of the cultivation period.
