# Supplementary material for: Radish and Spinach Seedling Production and Early Growth in Response to Struvite Use as a Phosphorus Source
Source: Plants (Basel). 2024 Oct 18;13(20):2917. doi: 10.3390/plants13202917 (PMC11511442; doi:10.3390/plants13202917)
Supplement: Supplementary file 1 [file plants-13-02917-s001.zip › plants-3237554-supplementary.pdf]

Table S1. Description of the treatments examined in the experiment.

Treatment	40-0-0+5S (mg L^{-1} substrate)	0-46-0 (mg L^{-1} substrate)	0-0-50 (mg L^{-1} substrate)	Struvite (mg L^{-1} substrate)	Fertigation solution (dS m^{-1})
NoFert	-	-	-	-	-
Fert	375	152	400	-	-
ST1	279	-	391	687	-
ST2	279	-	391	1374	-
NoFert+S	-	-	-	-	1.5 – 1.8
Fert+S	375	152	400	-	1.5 – 1.8
ST1+S	279	-	391	687	1.5 – 1.8
ST2+S	279	-	391	1374	1.5 – 1.8

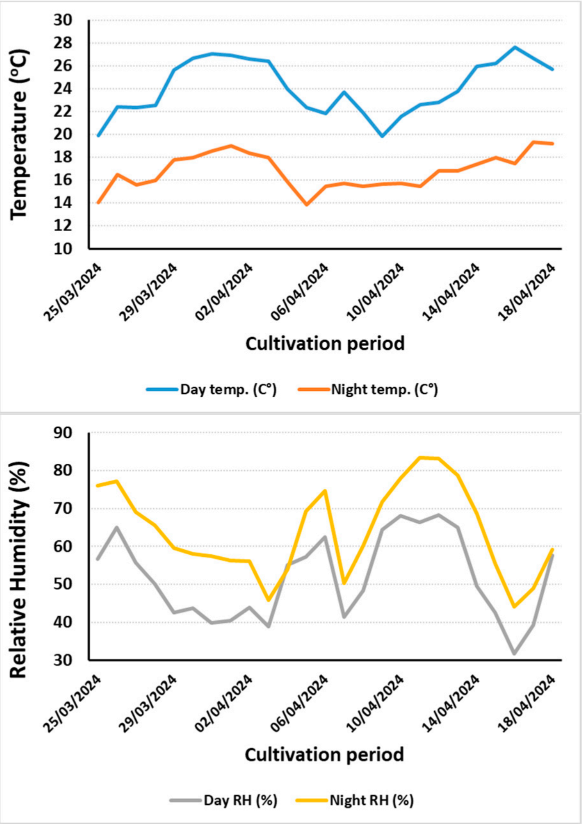

Figure S1. Average day and night temperature and relative humidity during the growing period.

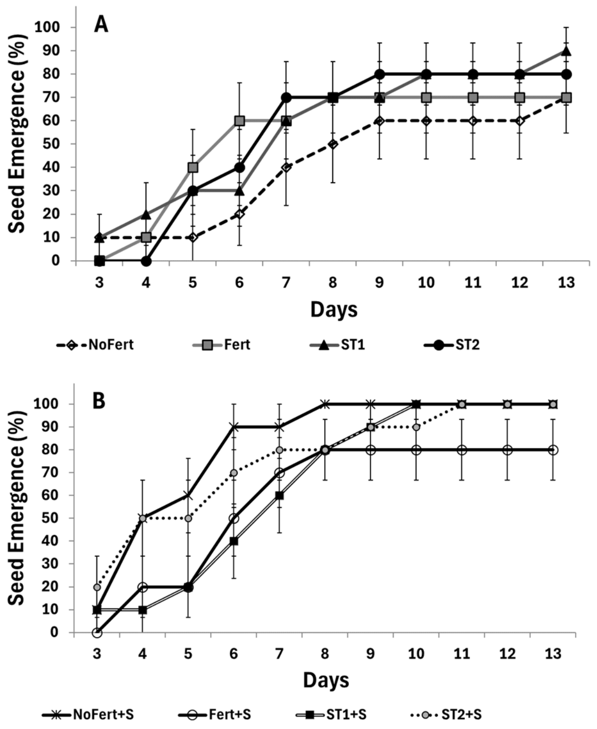

Figure S2. The effect of fertilizer application (NoFert, Fert, ST1, and ST2) and supplementary fertigation (+S) on daily seedling emergence (A, B) of radish seeds.

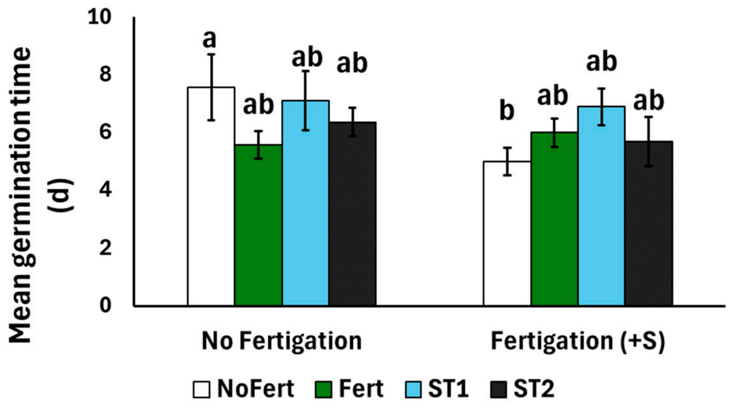

Figure S3. The effect of fertilizer application (NoFert, Fert, ST1, and ST2) and supplementary fertigation (+S) on mean germination time (d) of radish seeds.

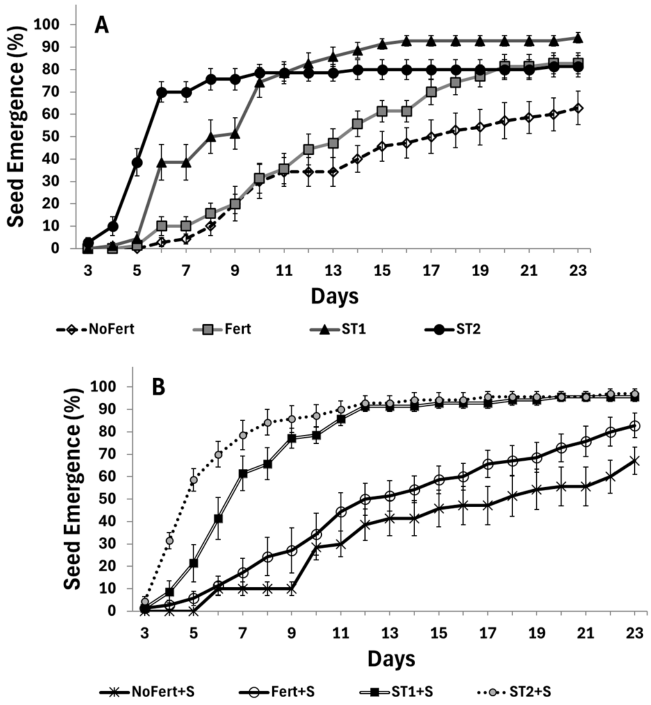

Figure S4. The effect of fertilizer application (NoFert, Fert, ST1, and ST2) and supplementary fertigation (+S) on daily seedling emergence (A, B) of spinach seeds.

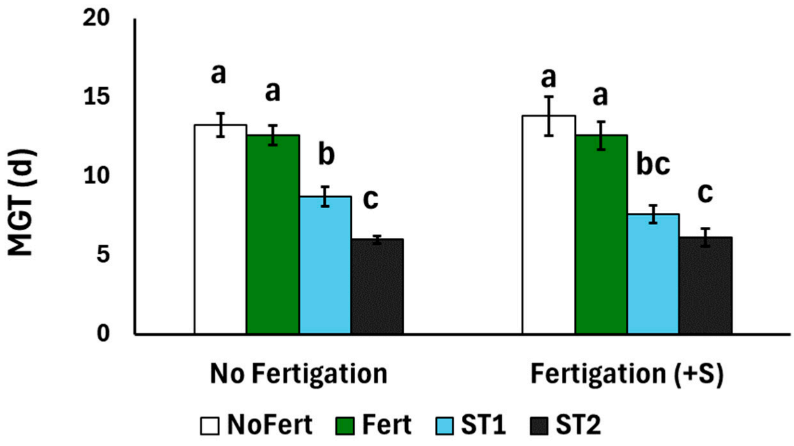

Figure S5. The effect of fertilizer application (NoFert, Fert, ST1, and ST2) and supplementary fertigation (+S) on mean germination time (d) of spinach seeds.

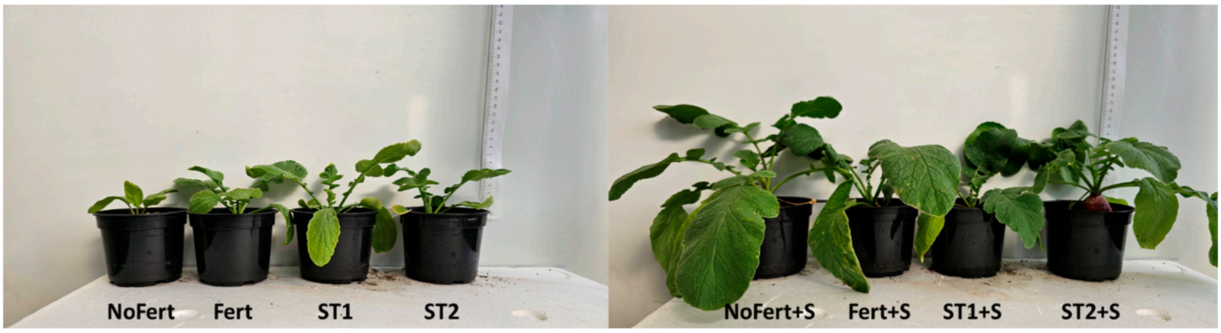

Figure S6. Photo comparison of radish growth between the examined treatments at end of the cultivation period

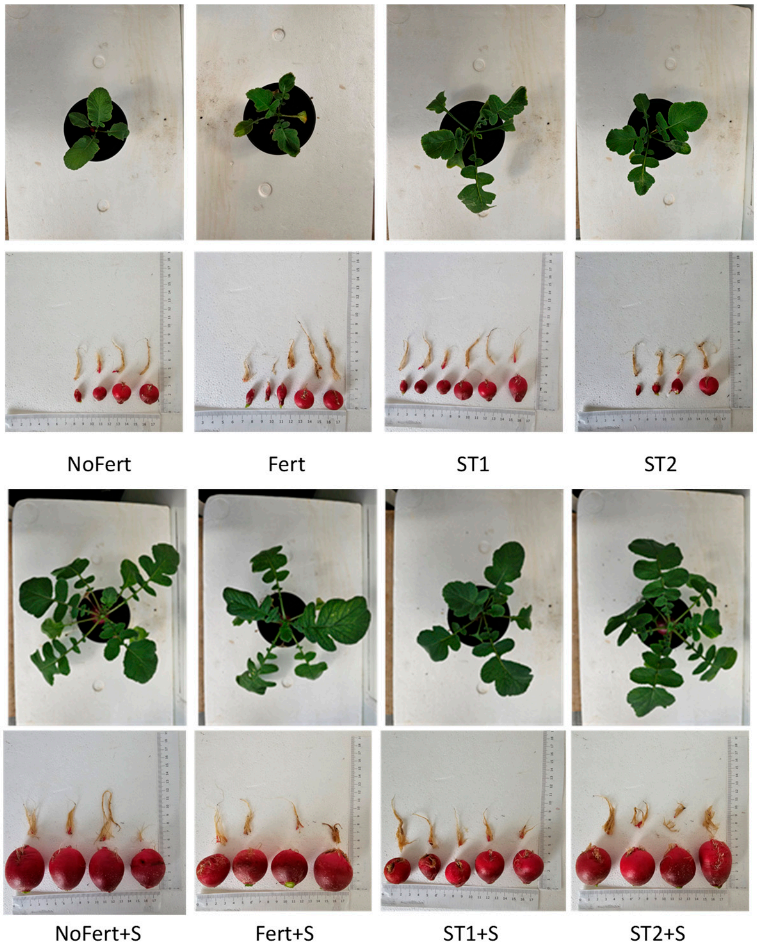

Figure S7. Photo comparison of radish growth between the examined treatments at end of the cultivation period

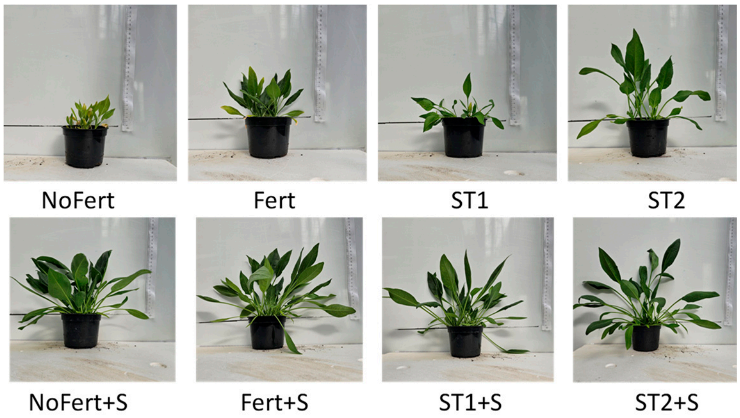

Figure S8. Photo comparison of spinach growth between the examined treatments at end of the cultivation period
